# Identification of risk factors for morbidity and mortality after Hartmann’s reversal surgery – a retrospective study from two French centers

**DOI:** 10.1038/s41598-020-60481-w

**Published:** 2020-02-27

**Authors:** Niki Christou, Thibaud Rivaille, Charlotte Maulat, Abdelkader Taibi, Fabien Fredon, Stephane Bouvier, Anne Fabre, Sophiane Derbal, Sylvaine Durand-Fontanier, Denis Valleix, Joan Robert-Yap, Fabrice Muscari, Muriel Mathonnet

**Affiliations:** 1 Service de chirurgie digestive, endocrinienne et générale, CHU de Limoges, Avenue Martin Luther King, Limoges Cedex 87042 France; 2 Chirurgie digestive et transplantation d’organes (département), Pôle digestif, Hôpital Rangueil, 1, avenue du Professeur Jean Poulhès - TSA 50032, 31059 Toulouse, cedex 9, France; 3 Department of Visceral Surgery, Geneva University Hospitals and Medical School, Geneva, Rue Gabrielle Perret-Gentil 4, 1205 Geneva, Switzerland

**Keywords:** Gastroenterology, Colon

## Abstract

Hartmann’s reversal procedures are often fraught with complications or failure to recover. This being a fact, it is often difficult to select patients with the optimal indications for a reversal. The post-recovery morbidity and mortality rates in the literature are heterogeneous between 0.8 and 44%. The identification of predictive risk factors of failure of such interventions would therefore be very useful to help the practitioner in his approach. Given these elements, it was important to us to analyze the practice of two French university hospitals in order to highlight such risk factors and to allow surgeons to select the best therapeutic strategy. We performed a bicentric observational retrospective study between 2010 and 2015 that studied the characteristics of patients who had undergone Hartmann surgery and were subsequently reestablished. The aim of the study was to identify factors influencing morbidity and postoperative mortality of Hartmann’s reversal. Primary outcome was complications within the first 90 postoperative days. 240 patients were studied of which 60.4% were men. The mean age was 69.48 years. The median time to reversal was 8 months. 79.17% of patients were operated as emergency cases where the indication was a diverticular complication (39.17%). Seventy patients (29.2%) underwent a reversal and approximately 43% of these had complications within the first 90 postoperative days. The mean age of these seventy patients was 61.3 years old and 65.7% were males. None of them benefited from a reversal in the first three months. We identified some risk factors for morbidity such as pre-operative low albuminemia (p = 0.005) and moderate renal impairment (p = 0.019). However, chronic corticosteroid use (p = 0.004), moderate renal insufficiency (p = 0.014) and coronary artery disease (p = 0.014) seem to favour the development of anastomotic fistula, which is itself, a risk factor for mortality (p = 0.007). Our study highlights an important rate of complications including significant anastomotic fistula after Hartmann’s reversal. Precarious nutritional status and cardiovascular comorbidities should clearly lead us to reconsider the surgical indication for continuity restoration.

## Introduction

**Currently**, Hartmann’s interventions are predominantly used as an emergency procedure due to general precarious conditions, such as septic shock but also in planned situations for elderly or frail patients with several comorbidities. Complicated diverticulitis and perforation or obstruction of colorectal cancers are the two major indications of this kind of surgery^1,2^. Hartmann’s reversal has been linked to high rates of complications. The review of Toro *et al*., analyzed the results of 30 studies where 684 patients benefited from a laparoscopic Hartmann’s reversal. They found a complication rate of 16.1% with significant heterogeneity between observations^3^. It is worth noting that even today, Hartmann’s reversal rates remain low, around 30%, showing no augmentation over the last 10 years^4^.

As a result, the reversal of this procedure remains a challenge for the surgeon to avoid complications in a context of inflammation, sepsis and/or several comorbidities already presenting with the patient. Therefore, it is crucial to carefully select the best timing and indication for this procedure. The surgeon must be able to carefully assess the patient’s eligibility for a secondary recovery of digestive continuity. He must also evaluate when the best moment to bring the patient back to the operating room would be in order to limit the complications so often associated with such a surgery.

Given the multiplicity of situations encountered, it seemed important to analyze the practice of two university hospitals and look at outcomes based on the decisions for reversal.

The purpose of this study was to identify risk factors influencing postoperative morbidity and mortality of Hartmann’s reversal and to propose a practical management scheme.

## Material and Methods

This bicentric study was conducted in two French university hospitals: Dupuytren Hospital (CHU Limoges) and Rangueil Hospital (CHU Toulouse). All methods were carried out in accordance with relevant guidelines and regulations. Protocol was approved by a named institutional local committee of University Hospital of Limoges. All patients were informed of such a study and gave informed consent.

In this retrospective observational study we included patients older than 18 years, corresponding to the French common classification of medical acts (CCAM [Classification Commune des Actes Médicaux] coding HHFA014, HHFA024 and HHFC040. These include different types of left colectomies without restoration of continuity (without release of the left colic angle by laparotomy, with release of the left colic angle by laparotomy, and without release of the left colic angle by laparoscopy), and have been operated on between January 2010 and December 2015.

Hartmann’s intervention is defined as the removal of a left colonic segment, associated with the abandonment of the sutured rectal stump in the abdomen, and the creation of a terminal colostomy on the upstream colonic segment.

Patients who had colectomy with double colostomy were not included in this study.

The decision to restore the digestive continuity was made between the patient and the surgeon according to the comorbidities, the initial pathology and the wish of the patient. The reversal procedure consisted of closure of the colostomy, dissection of the rectal stump and the creation of a colorectal anastomosis. Operative modalities, type of anastomosis and technique, were variable depending on the surgeon’s training. Post-operative care was provided with conventional hospitalization on the ward, continuing care or resuscitation according to clinical evolution.

Patients who did not benefit from reversal were those who had their original Hartmann’s procedure performed in another hospital over 20 months ago. This delay period was chosen because almost all of the reversals were completed within this time frame. Patients who were operated at another center were not included.

Primary outcome was the complication rate within 90 postoperative days.

Mortality, overall morbidity, severe morbidity (Dindo-Clavien ≥ 3), surgical morbidity and factors influencing the occurrence of anastomotic fistula were also studied.

### Data collection

Data was collected using internal software at Crossway (CHU Limoges) and Orbis (CHU Toulouse) hospitals. Missing data was retrieved from the handwritten records.

The data collected focused on these main points:

1. The pre-operative characteristics of the patients: (age, gender, BMI, comorbidities)
2. The patient’s disease (primary diagnosis, malignancy or non-malignancy of the pathology, emergency management or not)
3. The intraoperative data (surgeon’s experience, approach, type of anastomosis)
4. The postoperative data (mortality at day 30, morbidity at day 90, hospitalization area and length of hospital stay).

### Statistical analysis

The data were compiled into a Microsoft Excel® table (Microsoft Corporation, Santa Rosa, USA).

Statistical analyses were performed with SPSS® software for Windows v.22 (IBM, Chicago, USA).

Most of the variables collected were qualitative. Pre, per and post-operative epidemiological and clinical characteristics were compared with a chi-square test when enrolment permitted, or by Fisher’s exact test. The quantitative variables were compared by a Mann-Whitney U test. Multivariate analyses were performed by binary logistic regression. The criteria of inclusion of variables from the univariate analysis for the multivariate analysis was their statistical significance in the univariate analysis.

Some quantitative variables were treated in subcategories (albumin, BMI, age, hemoglobin) and analyzed as qualitative variables. A value of p <0.05 was considered significant.

## Results

During this period, 186 patients underwent surgery with a Hartmann’s procedure at Limoges University Hospital and 78 at Toulouse University Hospital, for a total of 264 patients (characteristics summarized in Table 1).

**Table 1:** Baseline demographic characteristics. Comparison of patients who had a Hartmann procedure in the two University centers.

Variable	Surgical department of Limoges N = 166 (69%)	Surgical department of Toulouse N = 74 (31%)	Total N = 240	P value
Sex (male)	100 (60%)	45 (61%)	145 (60.5%)	0.934
Age	69.3	69.8	69.5 (±14)	0.642
BMI (Kg/m^2^)	25.6	24.7	25.3 (±4.8)	0.109
ASA score ≥ 3	81 (49%)	39 (53%)	120 (50%)	0.576
Current smoker	72 (43%)	42 (57%)	114 (47.5%)	0.055
Alcohol	9 (5.4%)	5 (6.7%)	14 (5.8%)	0.443
Diabetes	32 (19%)	6 (8.1%)	38 (15.8%)	0.05
CKD (GFR < 60 ml/mn)	18 (11%)	15 (20%)	33 (13.8%)	0.05
Dialysis	1 (0.6%)	2 (28%)	3 (1.3%)	0.226
Hypertension	116 (69%)	40 (63%)	162 (67.5%)	0.238
CAD	44 (26.5%)	24 (32%)	68 (28.3%)	0.347
PAD	15 (9%)	4 (5%)	19 (7.9%)	0.336
COPD	24 (14.5%)	12 (16%)	36 (15%)	0.725
Liver cirrhosis	1 (0.6%)	0	1 (0.4%)	0.692
Digestive cancer	82 (49%)	33 (45%)	115 (48%)	0.492
Steroids	16 (10%)	11 (15%)	27 (11%)	0.237
Immunosuppressive	14 (8%)	5 (7%)	19 (8%)	0.657
drugs	24 (14%)	20 (27%)	44 (18.3%)	0.02
Warfarin	3 (2%)	0	3 (1.3%)	0.329
DOA	138 (83%)	52 (70%)	190 (79%)	0.023
Emergency procedure	165 (99%)	68 (92%)	233 (97%)	0.004
Laparotomy	102 (61.5%)	29 (39%)	131 (54.6%)	0.001
First surgeon senior				

ASA. American Society of Anesthesiologists; BMI. Body Mass Index CAD. coronary artery disease; COPD. Chronic obstructive pulmonary disease; CKD. chronic kidney disease; DOA. direct oral anticoagulant; GFR. glomerular filtration rate; PAD. peripheral arterial disease.

Patients who died as a result of the first intervention were excluded from this population secondarily (n = 24).

**Figure 1:**
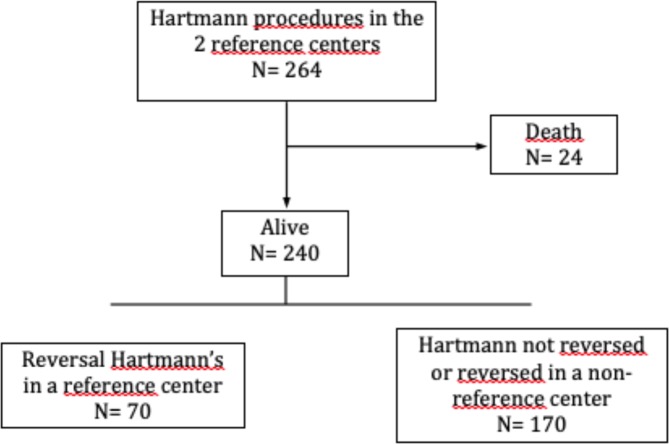
Flowchart of patients operated on a reversal Hartmann’s.

Of the 240 patients who survived the Hartmann’s procedure, 70 of them benefited from an intervention aimed at restoring digestive continuity by colorectal anastomosis (Fig. 1): 15 at Limoges and 55 at Toulouse. The indications for the original Hartmann’s procedure in these 70 patients were: diverticulitis, cancer, with either occlusion or perforation, post-ischemia complications, chronic inflammatory bowel disease, carcinomatosis and contact invasion, urinary or gynaecological fistula, cancer without symptoms, volvulus, or palliative surgery. The average age of these patients was 61.83 years (standard deviation 12.34) of which 46 (65.7%) were male. The median recovery time was 7 months. (3–20) No patients benefited from reversal in the first three months; 58 (78.57%) patients underwent it in the first year. No reversal was achieved after 21 months. This delay was statistically shorter in case of non-neoplastic pathology 7.38 (3–20) versus 11.26 (6–18) months (p < 0.0001).

The intervention was performed by a senior surgeon in 50 cases (71.3%) and the other cases by a junior surgeon.

The approach was by laparotomy in 68 patients (97.14%), of which 6 procedures had been started laparoscopically but converted to an open surgery due to technical difficulties. Reversal was completely performed under laparoscopic control in 2 cases (2.86%). The anastomosis was done by mechanical stapling in 44 cases (62.9%) and by manual suture in 24 cases. In 57 cases (81.4%) it was latero-terminal anastomosis and termino-terminal in 11 cases (18.6%). A protective stoma was performed in 6 cases (8.57%), 3 ileostomies and 3 colostomies (Table 1).

Three patients (4.29%) died within 30 days postoperatively and 30 patients (42.8%) had a postoperative complication within 90 days. These complications were medical in 23 cases (32.86%) and surgical in 13 cases (18.57%) including 4 anastomotic fistulas (5.7%) requiring 3 surgical revisions (4.29%). Among the 6 patients with protective ostomy in addition to the Hartmann reversal, two complications were present; acute renal failure and medically treated recto-vaginal fistula that progressed to stenosis.

Only 5 patients had severe complications as defined by a Dindo-Clavien score ≥ 3. The average length of stay was 13.34 days (± 8.24 days). For these patients, an invasive procedure was required: 2 needed radiologic drainages of pelvic abscesses with fistula, 1 had another Hartmann for peritonitis with fistula, 1 had a laparostomy for indeterminate peritonitis and 1 required abscess drainage by laparotomy including a small bowel suture to close a small bowel wound. Overall outcomes are presented in Table 2.

**Table 2:** Overall outcomes of patients who had a Hartmann reversal procedure.

Outcomes	N = 70
Reversal procedure time. months. median (range)	7 (3–20)
First surgeon senior. n (%)	50 (71.3%)
Stapled anastomosis. n (%)	44 (62.9%)
Defunctioning stoma. n (%)	6 (8.5%)
Intensive care unit stay. n (%)	60 (85%)
Hospital stay. d. median (range)	13 (5–21)
Mortality. n (%)	3 (4.2%)
Overall morbidity. n (%)	30 (42.8%)
Mild Complications: Clavien-Dindo I-II	25 (35.7%)
Serious complications: Clavien-Dindo III-IV-V	5 (7.1%)

Analysis of postoperative complications (Table 3) identified hypoalbuminemia as a significant factor in the postoperative complication group, (29.58 g/L versus 36.66 g/L, p = 0.005) (Table 4). Patients with albumin levels ≤ 35 g/L, corresponding to moderate malnutrition, were exposed to a higher postoperative morbidity (29.17% vs 70.83%, p = 0.002). Surgical morbidity was also significantly increased by the presence of obesity (BMI 25–30, p = 0.032) (Table 4). Using a composite score such as Charlson score, there was no statistical significance of morbidity or mortality (Tables 5 and 6**)**. After multivariate analysis, only albumin rate ≤ 35 g/L increased post operative morbidity (p = 0.007) and there was just a tendency of increase with obesity (BMI 25–30, p = 0.642) (Table 7).

**Table 3:** Data of the 90-days perioperative complications (some patients had more than one complication).

Postoperative complication	N (%)
Medical complications	23
Cardiopulmonary disease	8 (11.4%)
Acute kidney failure	4 (5.7%)
Urinary infection	5 (7.1%)
Sepsis (not linked to the surgery)	4 (5.7%)
Others	2 (2.8%)
Complications related to the surgery	18
Wound infections	6 (8.5%)
Sepsis (deep abscess)	3 (4.2%)
Peritonitis	2 (2.8%)
Fistula	4 (5.7%)
Hemorrhage	3 (4.3%)
Unplanned surgical reoperation	3 (4.3%)

**Table 4:** Univariate analysis of complications after Hartmann reversal procedure (HRP).

Variable	No complications N = 40	Complications N = 30	Total N = 70	P value
Sex				
Male	25	20	45	0.72
Female	15	10	25	
Age.				
[28–50]	8	4	12	0.464
[51–70]	25	15	40	0.295
[71–90]	7	11	18	0.07
BMI (Kg/m2)	26 (20–33)	26 (20–47)	26 (20–47)	0.43
BMI 18.5–25	32	17	13	0.158
**BMI 25–30**	**23**	**6**	**24**	**0.032**
BMI >30	13	7	33	0.432
ASA score ≥3	11	11	22	0.41
Current smoker	18	14	32	0.89
Alcohol (>30 g/day)	20	4	5	0.1
Diabetes	8	3	11	0.21
CKD (GFR <60 ml/mn)	1	3	4	**0**.181
Hypertension	21	19	40	0.365
CAD	2	2	4	0.58
PAD	2	4	6	0.21
COPD	3	5	8	0.2
Digestive cancer	15	10	25	0.72
Steroids	5	3	8	0.53
Immunosuppressive drugs	3	2	5	0.64
Warfarin	3	2	5	0.64
Time to get reversal (months)	7.9 (3–20)	8.3 (3–18)	8.1 (3–20)	0.53
First surgeon senior	30	20	50	0.445
Median operative time (mn)	196 (65–600)	218 (60–650)	206 (60–650)	0.77
Manual anastomosis	15	9	24	0.65
Diverting stoma	4	2	6	0.48
**Albumin (g/l)**	**36 (26–44)**	**29 (20–42)**	**33 (20–44)**	**0.005**
**Albumin ≤ 35 g/l**	**7**	**17**	**24**	**0.002**
Hemoglobin (g/dl)	13 (10–16)	12.6 (9–15)	13 9–16)	0.124
Length of stay (days)	15 (7–32)	26 (9–63)	21 (7–63)	<0.0001

BMI. Body Mass Index; CAD. coronary artery disease; COPD. Chronic obstructive pulmonary disease; CKD. chronic kidney disease; DOA. direct oral anticoagulant; GFR. glomerular filtration rate; PAD. peripheral arterial disease.

**Table 5:** Univariate analysis of complications after Hartmann reversal procedure (HRP) according to Charlson Score.

	Charlson < 3	Charlson ≥ 3	P value
Overall Morbidity	9/70 (12.86%)	21/70 (30%)	0.388
Mortality	0/70 (0%)	3/70 (4.28%)	0.187
Medical Complication	7/70 (10%)	16/70 (22.86%)	0.519
Surgical Complication	4/70 (5.71%)	9/70 (12.86%)	0.68

**Table 6:** Univariate analysis of Clavien-Dindo classification of complications after Hartmann reversal procedure (HRP) according to Charlson Score.

	Charlson < 3	Charlson > egal 3	p
Dindo 345	1/25 (4%)	4/45 (8.89%)	0.447
Dindo 12	8/25 (32%)	17/45 (37.78%)	0.629

**Table 7:** Multivariate analysis for complications after Hartmann reversal procedure (HRP).

Variables	OR	IC 95%	p
IMC 25–30	0.60	[0.069–5.188]	0.642
Alcohol > 30 g/j	5.5 × 10^8^	[0.000 –.]	0.999
**Albumin < 35 g/l**	11.95	[1.985–71.972]	**0.007**

Four anastomotic fistulas developed, which represents 5.7% of the cases. Their occurrence was related in univariate analysis **(**Table 8**)** to the presence of coronary artery disease (CAD) (p = 0.014), moderate chronic renal failure (p=0.014) and corticosteroid use (p = 0.004). This complication was statistically related to an increase in both length of stay and postoperative mortality (Table 8). To eliminate potentially confusing factors, a multivariate analysis was made but did not demonstrate any statistically significant criteria (just a tendency) as a risk factor for anastomotic fistula (CAD, p = 0.084; CKD, p = 0.084; steroid use, p = 0.059) (Table 9).

**Table 8:** Univariate analysis. risk factors for anastomotic fistula (n = 4).

Variable	Total	Anatomotic fistula	No anastomotic fistula	p
Age (years)	61.82 (28–90)	63.25 (52–68)	61.74 (28–90)	0.836
[28–50]	12 (17.14%)	0	12	0.3
[51–70]	40 (57.14%)	4 (10%)	36 (90%)	0.08
[71–90]	18 (25.71%)	0	18	0.42
Men	45 (64.3%)	4	41	0.162
Women	25(35.7%)	0	25	
BMI (Body Mass Index)(Kg/m^2^)	26.33 (19.72–47.27)	24.46 (20.2–29.39)	26.45 (19.72–47.27)	0.472
ASA ≥ 3	22 (31.3%)	3	19	0.89
Current smoker	32 (45.71%)	3	29	0.245
Alcohol	5 (7.14%)	1	4	0.262
Diabetes	11 (15.71%)	1	10	0.504
Obstructive chronic brochopneumopathia	8 (11.43%)	0	8	0.608
High blood pressure	40 (57.14%)	4	36	0.1
**Coronary artery disease**	**4 (5.71%)**	**2**	**2**	**0.014**
Arteriopathy obliterating lower limbs	6 (8.57%)	1	5	0.307
**Renal failure < 60 ml/min MDRD**	**4 (5.71%)**	**2**	**2**	**0.014**
History of neoplasia	25 (35.7%)	1	24	0.55
History of chemotherapy	17 (24.29%)	0	17	0.32
Pelvic radiotherapy	2 (2.86%)	0	2	0.888
**Corticosteroids**	**8 (11.43%)**	**3**	**5**	**0.004**
Immunosuppressives	5 (7.14%)	1	4	0.26
Warfarin	5 (7.14%)	1	4	0.26
Benign pathology	55 (78.57%)	4	51	0.37
Reversal time (months)	8.16 (3–20)	8.75 (3–20)	8.12 (3–18)	0.652
Senior surgeon	50 (71.43%)	2	48	0.32
Operating time (min)	205.4 (60–650)	211.25 (165–300)	205 (60–650)	0.437
Manual anastomosis	24 (35.29%)	1	23	0.56
Peritoneal anastomosis	9 (12.86%)	1	8	0.44
Rectal cut	32 (45.71%)	3	29	0.245
Stoma of protection	6 (8.571%)	0	6	0.69
Albumin (g / l)	33.12 (20.6–44)	27.85 (20.7–35)	33.41 (20.6–44)	0.344
Hemoglobin (g / dl)	13.01 (9.1–16.2)	12.7 (10.6–15.6)	13.03 (9.1–16.2)	0.624
**Death**	**3 (4.29%)**	**2**	**1**	**0.007**
**Length of stay (days)**	**13.34 (7–57)**	**34.25 (12–57)**	**12.08 (7–38)**	**0.04**

**Table 9:** Multivariate analysis for anastomic fistula.

Variables	OR	IC 95%	p
CAD (Coronary artery disease)	22.056	[0.661–735.8]	0.084
CKD (GFR <60 ml/mn)	22.056	[0.661–735.8]	0.084
Steroids	16.53	[0.896–304.8]	0.059

CKD. chronic kidney disease.

As a result, uncorrected hypoalbuminemia seems to be a contraindication to a Hartmann’s reversal; furthermore, obesity (BMI, 25–30), coronary artery disease, chronic renal disease and corticosteroid use should signal caution when considering a Hartmann’s reversal.

## Discussion

This study analyzed the complications and their predictive risk factors after Hartmann’s reversal interventions over a 6-year period in two French digestive surgery departments.

It is worth noting that none of the patients benefited from Hartmann’s reversal before 3 months after the surgery due to the patient’s general status or disease severity. Contrary to this, reversal of a protective lateral ileostomy before this delay never demonstrated consequences on morbidity^5^.

No statistical differences have been pointed out concerning complications with relation to the time of reversal contrary to the study of Fleming *et al*.^6^ which underlined more post reversal complications when time to reversal increased (44% complication rate if the Hartmann reversal is performed more than 9 months of the original surgery).

High overall morbidity rate (42.8%) after Hartmann’s reversal in this study (which included surgeries from 2010–2015) was similar to literature of the 2000s^7^. It is worth noting that more recent studies have shown overall morbidity rates lower at approximately 22.7%^8^. However, this study of Chereau *et al*.^8^ encompassing similar indications for Hartmann’s reversal as our study, reported severe complication rates of 11.7% (Dindo 3–4). This is higher than the rate of 4.3% (Dindo 3–4) found in our bicentric study but similar to that of Hallam *et al*.^9^. These low results have to be taken with precaution as our work is retrospective and may have missed some failed complications of level Dindo 1–2, which are, most often managed as outpatients. More specifically, postoperative complications were dominated by wound infections (around 9%), which is similar to the recent work of Hallam *et al*.^9^.

Hospital stay sharply increased with complications (p < 0,0001). Albumin rate lower than 35 g/L lead to a significant rise in complications, in accordance with literature (p = 0.002)^10^.

Anastomotic fistula rate was found in 5.7% of the cases and corresponded to the rate usually found, between 2–7%^11^, with highest rates coming from older studies^12^. The risk factors identified in univariate analysis, long-term corticosteroid use, renal failure and coronary artery disease, reflect a precarious vascular state. Although no criterion was significant in multivariate analysis, there was a trend (p = 0.059) showing the use of steroids as a risk. This analysis on a larger cohort would allow a better estimate. Patients with anastomotic fistula in this study were at high risk of death (p = 0.007). Eveno *et al*.^13^ also showed a correlation between the rate of anastomotic fistula and postoperative mortality as a function of the degree of aortic calcification. The latter was evaluated by a method derived from the CT coro-scanner. It may be interesting to systematically order a preoperative CT according to this protocol, in order to identify patients at particular risk in advance. In our study, 100% of patients had received a CT during their care. Moreover, the use of CT may be important to evaluate nutritional status by measuring the psoas muscle and its lipid mass.

In contrast to the Hartmann’s procedure, the reversal is considered more technically complex and was more frequently performed by a senior surgeon (71.43 vs. 54.58%). It is essential to note that neither the operative time, the type of anastomosis, its height on the rectum or the surgeon’s experience was correlated with an increase in morbidity or postoperative mortality.

In our series, most of the interventions were performed as “open surgeries”. Recently, studies have focused on the interest of using laparoscopy. Most of them have shown lower morbidity and length of hospital stay^14,15^.

It is important also to underline that the recent development of new tools have improved the technical aspects of this surgery, especially for laparoscopy. For instance, the use of intraoperative perfusion of indocyanine green (ICG) with near-infrared (NIR) visualization can orient the selection of intestinal section level and assess anastomotic vascularisation^16^ for improved outcomes in anastomoses.

However, it is necessary to highlight that this study presented some drawbacks. Considering its methodology, it would be have been better to conduct a prospective study in order to avoid any missing data. As a result, the high proportion of missing data concerning albumin (22/70) calls for caution but should lead to a better pre-operative nutritional assessment, although it is currently recommended only in the context of cancerous pathology. Furthermore, the number of patients was not probably enough to figure out any predictive risk factor for reversal. Thus, CAD, chronic renal impairment and use of steroids, which showed just a tendency to increase overall morbidity, have to be taken into account in the general management. More precisely, concerning the risk factors associated with anastomotic fistula, the numbers to compare were too low (4 patients versus 66). An impact of fistula in terms of death and length of hospital stay can be seen. CAD and steroids seem also to be associated with fistula as previously described in literature. However, multivariate analysis for these low numbers cannot be taken into account.

## Conclusion

This study shows a significant morbidity of 42.8% and a high mortality (4.29%) frequently related to the occurrence of an anastomotic fistula (5.7%).

We have shown a significant increase of overall postoperative morbidity with low albumin levels. However, the power of our study was not sufficient enough to demonstrate this for obesity, CAD, moderate chronic renal failure and long-term corticosteroids use. All these factors reflecting an altered general state should invite physicians to carefully consider the surgical indication for a Hartmann’s reversal. Finally, we should focus on taking time to systematically improve nutritional status and overall general status, as this may help reduce patient’s risk of developing complications after a Hartmann’s reversal.
